# International trends and social disparities in pain of adults aged 50 years and older in 22 countries across Europe, Asia, and the Americas: a longitudinal population-based study

**DOI:** 10.1016/j.lanhl.2025.100808

**Published:** 2025-12-31

**Authors:** Esteban Calvo, Jose T Medina, Hanna Grol-Prokopczyk, Katherine Keyes, Alvaro Castillo-Carniglia, Antonia Díaz-Valdés, Tamara Otzen, Robin Richardson, Silvia Martins

**Affiliations:** **Society and Health Research Center, Universidad Mayor, Santiago, Chile** (Prof E Calvo PhD, J T Medina MS, A Díaz-Valdés PhD); **School of Business, Faculty of Social Sciences and Arts, Universidad Mayor, Santiago, Chile** (Prof E Calvo); **Millennium Nucleus for the Evaluation and Analysis of Drug Policy (nDP), Santiago, Chile** (Prof E Calvo, Prof A Castillo-Carniglia PhD); **Department of Sociology and Criminology, University at Buffalo, New York, NY, USA** (H Grol-Prokopczyk PhD); **Mailman School of Public Health, Columbia University, New York, NY, USA** (Prof K Keyes PhD, Prof S Martins PhD); **Departamento Nacional de Salud Pública, Facultad de Medicina, Universidad San Sebastián, Santiago, Chile** (Prof A Castillo-Carniglia); **Doctorado en Ciencias Médicas, Universidad de la Frontera, Temuco, Chile** (T Otzen PhD); **Rollins School of Public Health, Emory University, Atlanta, GA, USA** (R Richardson PhD)

## Abstract

**Background:**

The experience of pain is highly prevalent among older adults worldwide. This study aimed to compare trends and social disparities in pain for middle aged and older adults (ie, those aged ≥50 years) across countries to help identify high-pain or high-disparity hotspots needing intervention, and low-pain or low-disparity locations or timepoints from which policy and practice lessons could be drawn.

**Methods:**

Country-specific pain trends were estimated using longitudinal logistic regressions on harmonised data for 212 904 adults aged older than 50 years observed repeatedly from 1998 to 2018 in 22 countries across three continents.

**Findings:**

Unadjusted pain prevalence was 43⋅21% (95% CI 43⋅10–43⋅33) across all countries and years pooled. Instrument-adjusted prevalence ranged from 26⋅70% (95% CI 25⋅52–27⋅88; Netherlands, 2006) to 59⋅20% (58⋅02–60⋅38; France, 2016). Over the decade 2006–16, when most countries were observed, prevalence substantially increased in 15 countries (12⋅01 percentage points), decreased in China (6⋅60 percentage points), and remained steady in six countries. Prevalence was higher among women, those with less education (ie, completed less than high school), and older respondents (those aged >60 years), though disparities varied substantially across countries. Disparities widened over time in some countries (eg, Spain), but remained stable, declined, or reversed in others (eg, Sweden). In 2016, Denmark had the lowest overall pain disparity index, while South Korea had the highest.

**Interpretation:**

There was a substantial increase in pain prevalence among middle aged and older adults in most countries studied. Cross-country, temporal, and sociodemographic variation indicates pain should be treated as a population public health issue.

## Introduction

The experience of pain is a major concern for individuals and societies.^1–3^ Pain is the leading cause of years lived with disability, and its prevalence surpasses that of cancer, diabetes, and heart disease combined.^4–7^ Pain is triggered by various conditions but is not merely an inevitable consequence of them or of ageing; it can be prevented and treated.^1,2^ In the USA, 2008 expenditure on adults’ chronic pain was 3⋅7–4⋅2% of gross domestic product, underscoring the resources required to address pain.^8^

However, little research exists on international trends and disparities (ie, systematic group differences) in pain among middle-aged and older adults (ie, those aged ≥50 years), who are more likely to experience pain than younger people (ie, those aged <50 years),^7,9–12^ and for whom chronic pain is a frequently diagnosed condition and a leading cause of disability, and a barrier to healthy ageing.^13,14^ Yet, pain is not routinely assessed in national surveys, limiting global surveillance.^15^ Cross-national research can identify regions with high pain levels or disparities, for which prevention, education, and treatment might be needed, as well as historical locations with low pain levels or disparities for which policies and practices could be studied and replicated. Documenting pain trends and disparities can clarify how population and policy changes shape pain outcomes. Studying international pain trends and disparities is crucial to broadening the understanding of pain from a solely individual-level predicament to a population health concern.

Few studies use nationally representative cross-national micro-data for older adults, with a notable exception being the examination of pain among older adults in Europe.^9^ Most studies use Global Burden of Disease aggregate estimates of specific conditions such as low back pain,^4–7^ crude Gallup estimates for pain felt yesterday,^3^ non-representative samples of nations or age groups,^16^ or single-country data to estimate pain prevalence or risk factors,^17^ reflecting a lack of comparable individual-level data on general (ie, not site-specific) pain across countries and over time. Among individuals aged 50 years and older, previous studies have documented a 2–3% average annual increase in pain prevalence between 1992 and 2014 in the USA^17^ and an average annual increase of 2⋅2% between 2004 and 2011 and of 5⋅8% between 2013 and 2015 in 12 of 15 European countries.^9^ However, other studies report small decreases in chronic pain prevalence (from 38⋅5% to 33⋅5%) between 2011 and 2013 among Swedish people aged 65 years and older,^18^ and in low back pain prevalence (from 8⋅2% to 7⋅5%) between 1990 and 2017 among the general world population, with regional differences.^4,19^ These mixed findings are difficult to synthesise due to heterogeneous measures and methods across single-country studies. Research using comparable cross-national data on pain is rare beyond low back pain,^7,15^ and to the best of our knowledge no previous study has compared trends in any pain among middle-aged and older adults across three continents.

Each country’s sex, socioeconomic class, and age patterns could shape pain trends and disparities.^1,7,10–12,15,20–23^ Population-based epidemiology consistently documents that:^23^ women report more pain than men,^7,20,24^ individuals with lower education levels report more pain than those with higher education,^21^ and pain is more prevalent in older than younger adults partly due to the accumulation of pain-producing injuries or diseases with age.^11^ Although well established, little literature examines how these disparities vary across countries. To the best of our knowledge, this study is the first cross-country longitudinal study using individual-level repeated measures of any pain in adults aged 50 years and older across three continents, extending beyond condition-specific estimates such as that of low back pain.^7,23^ We aimed to test for cross-national variability in pain prevalence trends among middle-aged and older adults and to compare disparities in pain by sex, class, and age across countries and over time.

## Methods

### Data and sample

Longitudinal data from 22 countries came from six nationally representative cohort studies (appendix p 2): the Survey of Health, Ageing, and Retirement in Europe (SHARE), the Health and Retirement Study (HRS), the English Longitudinal Study of Ageing (ELSA), the Mexican Health and Aging Study (MHAS), the China Health and Retirement Longitudinal Study (CHARLS), and the Korean Longitudinal Study of Aging (KLOSA). The dataset comprised 212 904 individuals aged 50 years and older, with comparable pain measures across countries and over time (appendix p 2). Using the Gateway to Global Aging Data as a guideline, we harmonised 1998–2018 pre-pandemic data, with 1⋅01% of data points missing and 19⋅16% attrition between waves.^1^ All non-missing data were analysed, but we plotted only 2006–16 data to avoid over-extrapolating, as few countries had data outside this period.

### Pain outcome

To obtain a comparable measure across surveys, countries, and years, we harmonised two types of pain questions into a dichotomy indicating any pain. General questions, used in CHARLS, ELSA, and HRS (Are you often troubled with pain?), MHAS (Do you often suffer from pain?), and, starting in 2013, in SHARE (Are you troubled with pain?), did not reference any body parts. Site-specific questions, used in KLOSA (In what part of your body do you feel pain?) and SHARE up to 2011 (Pain in your back, knees, hips, or any other joint), primed respondents to consider particular locations.

Because site-specific questions could yield higher prevalence,^9^ we created a dichotomous variable for site-specific versus general questions and included it in all models to isolate instrument-related differences. This approach follows established cross-national harmonisation standards and allows measurement heterogeneity to be modelled explicitly rather than confounding cross-country comparisons. Consistent with previous psychometric work, self-reported pain is widely used in epidemiology and clinical practice due to its feasibility, validity, and reliability across populations, and the absence of an objective biomarker. ^2,21,25^

Given that not all surveys explicitly measure chronic pain, we also calculated the percentage of respondents reporting pain in each wave who did so in the preceding or following wave (74%), indicating substantial continuity over time. More details on harmonisation and variable characteristics are provided elsewhere.^1^

### Independent variables

Our main covariates included country and survey year. We further separated participants by sex, education, and time-varying age. Sex was coded as female or male. Due to fewer highly educated individuals and older adults in some countries, education was coded as less than high school completed versus high school completed or more, while age was coded as young-old (ie, those aged 50–60 years) and older-old (ie, those aged >60 years).

### Statistical analysis

We first estimated raw total and country-specific pain prevalence (ie, baseline, last wave, and pooled) and average annual change (baseline to last wave). Next, we used random-effects longitudinal logistic regressions (a form of generalised linear mixed models) to model the unbalanced data structure, including all repeated observations of pain among individuals over time. Model 1 includes country, time, and their interaction, adjusting only for the type of question. Country fixed-effects account for unobserved time-invariant country characteristics. Models 2 to 4 explore social disparities in the international pain trends, including two-way interactions between sex (Model 2), education (Model 3), and age (Model 4) with both country and time. Model 5 focuses on a high-risk group of women aged >60 years with low levels of education.

In Models 1–5, we obtained predicted probabilities and plotted them by country and time. We focused on 2006–16 predictions to minimise extrapolation, as few countries had data outside this period. Standardised measures were obtained by holding covariates constant (ie, male, high school completed or more, and older than 60 years). Using 2016 predictions, we constructed a social disparity index by summing percentage point differences across sex, education, and age groups, and then mapped regional patterns.

Due to the high computational demands of panel logistic regressions, which cannot be parallelised across CPUs, we used unweighted two-way interactions and linear trends. Using Stata MP 17 on an 8-core, 3⋅2 GHz system, these estimations still took nearly two months.

### Role of the funding source

The funders had no role in study design, data collection, analysis, interpretation, writing, or submission for publication.

## Results

Pain prevalence across all timepoints and countries was 43⋅21% (297 035/687 369; 95% CI 43⋅10–43⋅33) and was higher for females, those with less education, and older respondents (table; country-specific demographic statistics are provided in the appendix p 3). 74% of respondents reporting pain also did so in the previous or following wave, indicating substantial continuity.

Regression models 1–5 included a dummy for site-specific (*vs* general) pain questions to account for instrument-induced bias (appendix pp 4–8). Priming respondents to consider pain in specific body parts resulted in pain prevalence of 14⋅56 percentage points higher than asking about pain in general (see appendix pp 9–13 for diagnostics and sensitivity analyses, supporting robustness).

**Figure 1:**
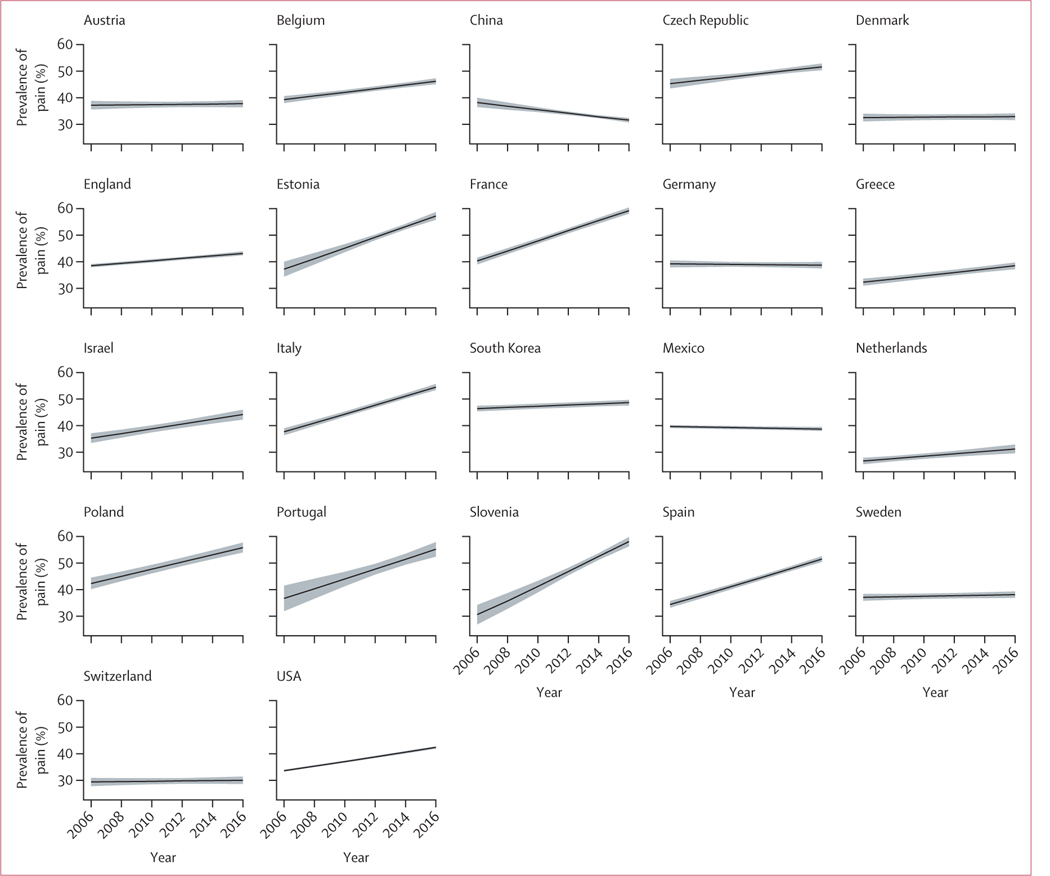
Pain prevalence in 22 countries, 2006–16 Shaded areas represent 95% CIs. Predicted prevalences based on model 1, estimated for site-specific pain question.

Using model-based predictions, we plotted country-specific predicted pain prevalence for 2006–16, when most countries were observed. The instrument-adjusted prevalence was lowest in the Netherlands in 2006 (26⋅70%; 95% CI 25⋅52–27⋅88) and highest in France in 2016 (59⋅20%; 58⋅02–60⋅38), with Slovenia showing the steepest growth (figure 1). Pain increased in 15 of 22 countries (as indicated in the appendix [p 14] by non-overlapping 2006 and 2016 95% CIs in Belgium, the Czech Republic, England, Estonia, France, Greece, Israel, Italy, South Korea, the Netherlands, Poland, Portugal, Slovenia, Spain, and the USA), with a mean annual increase of 1⋅21 percentage points, totalling 12⋅1 percentage points over a decade (from 37⋅11% in 2006 to 49⋅16% in 2016). Six countries showed negligible change (overlapping 95% CIs in Austria, Denmark, Germany, Mexico, Sweden, and Switzerland), while China experienced a significant 0⋅66 percentage point annual decline. These trends are unlikely to be fully explained by demographic shifts, as changes in sex composition, education levels, and population ageing were minimal over the observed period (appendix p 3).

Interaction terms in each model were significant as a group (p<0⋅0001 Wald test). We classified disparities as remaining stable if the gap changed by less than 1 percentage point over the decade (appendix p 15).

**Figure 2:**
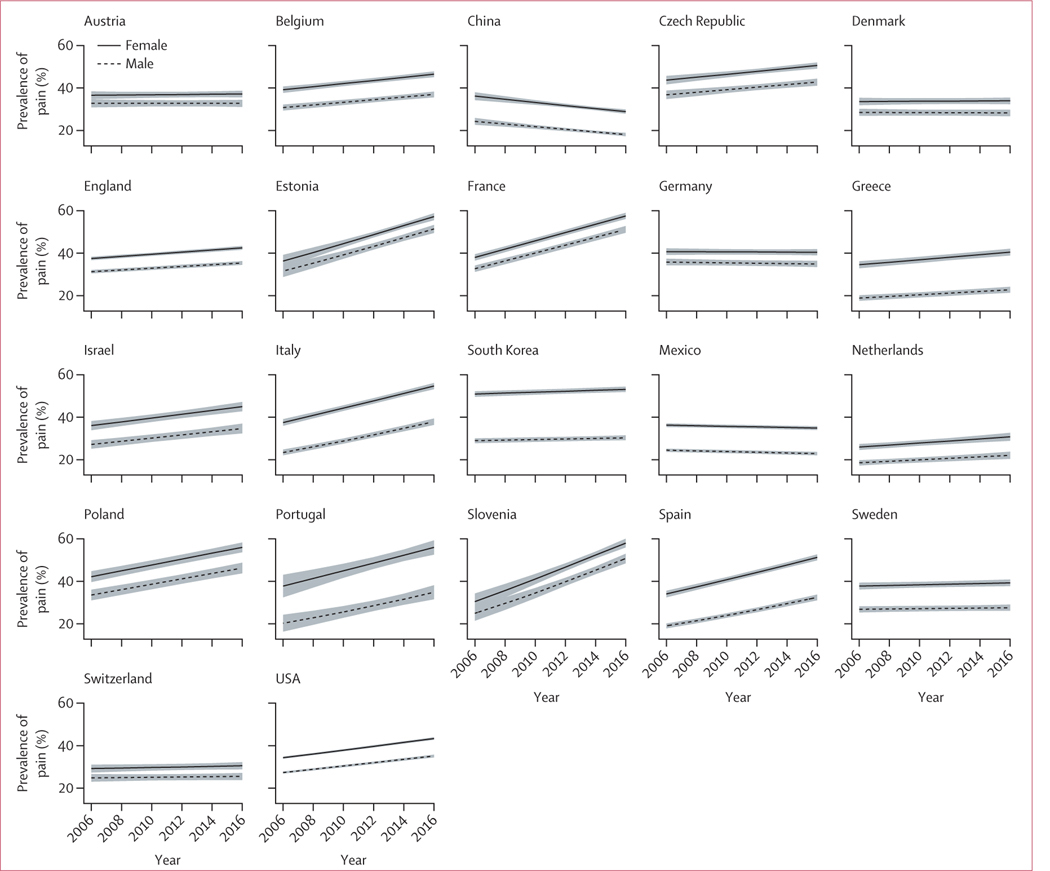
Pain prevalence by sex in 22 countries, 2006–16 Shaded areas represent 95% CIs. Predicted prevalences based on model 2, estimated for the site-specific pain question, high school completed or more, and the older-old (ie, those aged >60 years).

Females were more likely to report pain than males, with the size of this disparity varying substantially across countries (figure 2). The largest disparities (percentage points difference; p<0⋅05) were in South Korea 2016 (22⋅8; 95% CI 21⋅14 to 24⋅46), Portugal 2016 (21⋅1; 16⋅39 to 25⋅81), and Spain 2016 (18⋅8; 16⋅86 to 20⋅74). In 2006, sex disparities were not statistically significant in Estonia and Slovenia and were minimal in Austria (3⋅8; 1⋅16 to 6⋅44) and Switzerland (4⋅4; 1⋅76 to 7⋅04). Sex disparities showed little change over time in ten of 22 countries (Austria, Czech Republic, Denmark, England, France, Germany, South Korea, Mexico, Sweden, and Switzerland) and widened in 11 countries (Belgium, Estonia, Greece, Israel, Italy, the Netherlands, Poland, Portugal, Slovenia, Spain, and the USA), with the largest increases in Italy (2⋅7; –0⋅15 to 5⋅55), Portugal (3⋅6; –4⋅50 to 11⋅70), and Spain (3⋅8; 1⋅04 to 6⋅56), and a slight decrease in China (–1⋅1; –3⋅91 to 1⋅71).

**Figure 3:**
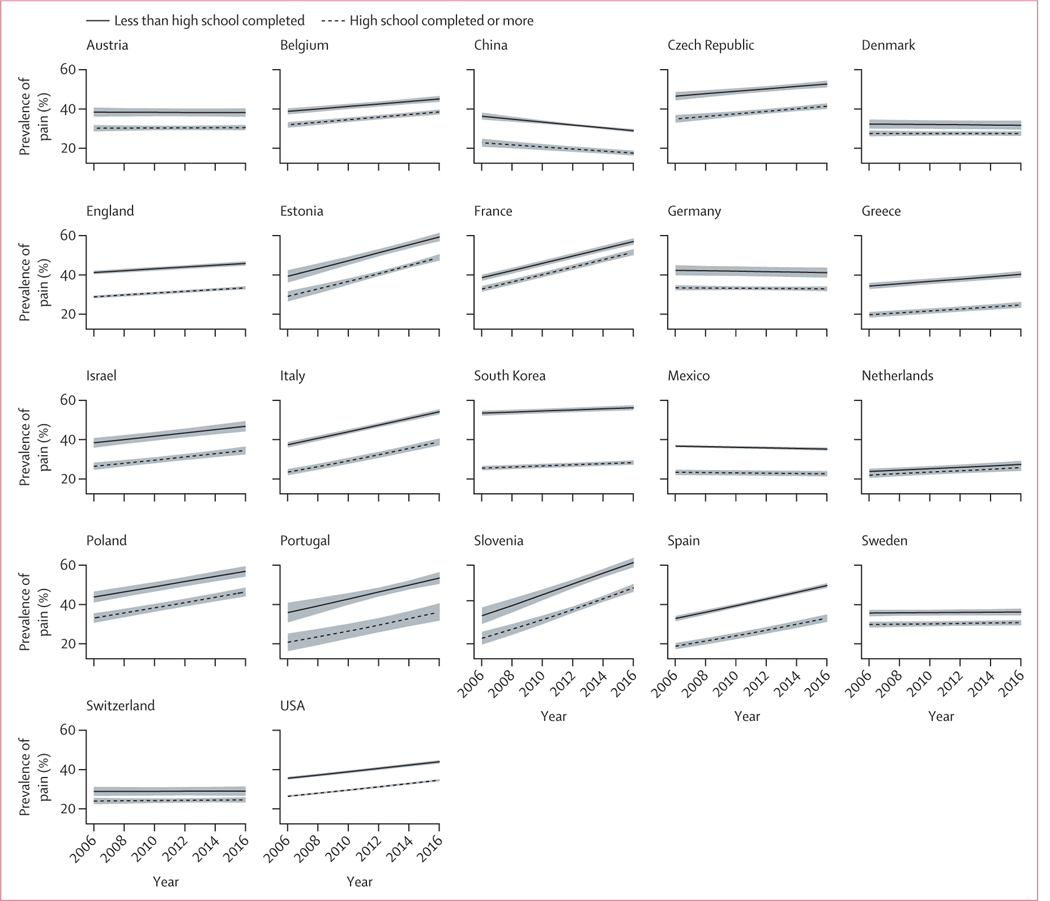
Pain prevalence by education in 22 countries, 2006–16 Shaded areas represent 95% CIs. Predicted prevalences based on model 3, estimated for the site-specific pain question, the older-old (ie, those aged >60 years), and males.

Respondents with less education were more likely to report pain than their counterparts, with the size of this disparity varying substantially across countries (figure 3). The largest disparities (percentage points difference; p<0⋅05) were in South Korea 2016 (27⋅9; 95% CI 26⋅24 to 29⋅56), Portugal 2016 (17⋅3; 11⋅92 to 22⋅68), and Spain 2016 (16⋅6; 14⋅31 to 18⋅89). Educational disparities were never significant in the Netherlands and were minimal in Denmark 2016 (4⋅2; 1⋅57 to 6⋅83) and Switzerland 2016 (4⋅4; 1⋅68 to 7⋅12). Educational disparities remained stable in 16 of 22 countries (Austria, Belgium, Czech Republic, Denmark, England, Estonia, France, Germany, Israel, South Korea, Mexico, the Netherlands, Poland, Sweden, Switzerland, and the USA), increased in five countries (Greece 1⋅0 [–1⋅95 to 3⋅95], Italy 1⋅4 [–1⋅66 to 4⋅46], Portugal 2⋅2 [–6⋅36 to 10⋅76], Slovenia 1⋅4 [–4⋅61 to 7⋅41], and Spain 2⋅5 [–0⋅59 to 5⋅59]), and decreased in China (–2⋅1; –5⋅17 to 0⋅97).

**Figure 4:**
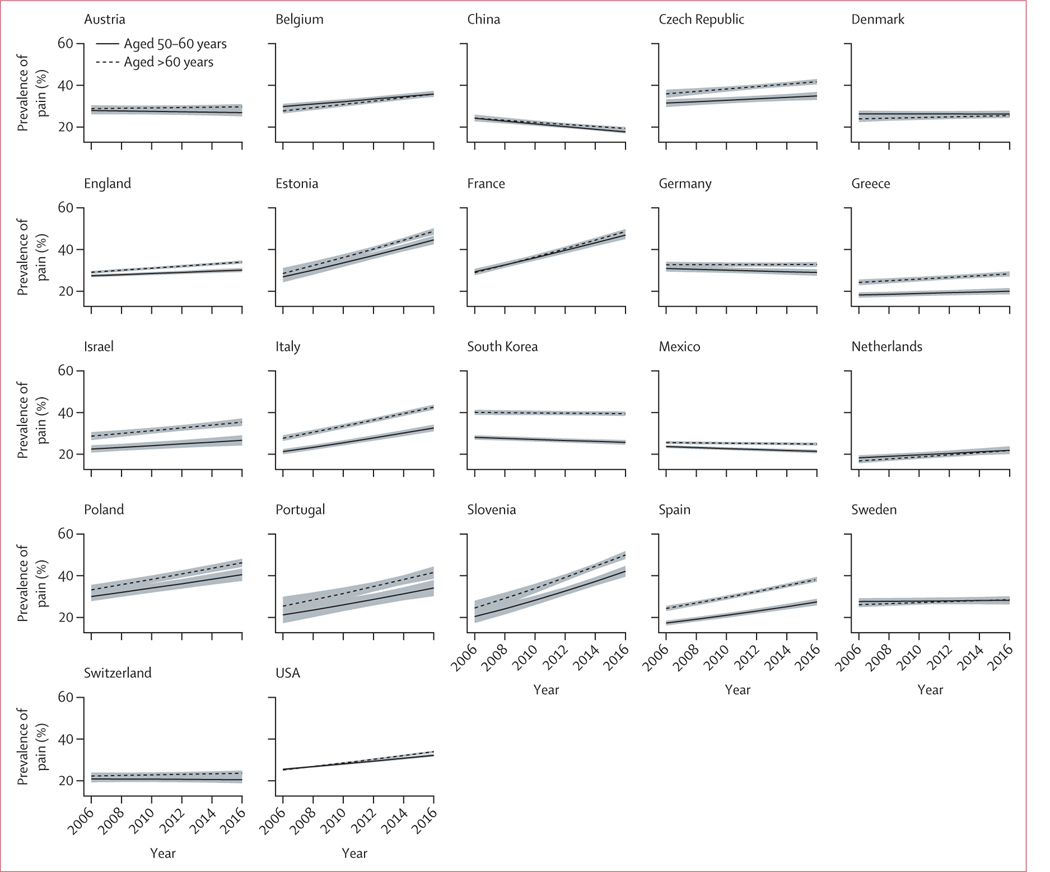
Pain prevalence by age group in 22 countries, 2006–16 Shaded areas represent 95% CIs. Predicted prevalences based on model 4, estimated for the site-specific pain question, high school completed or more, and males.

The direction of age disparities in pain was not consistent across countries (figure 4). Most often, the older-old were more likely to report pain than the younger-old (Czech Republic, England, Estonia, Germany, Greece, Israel, Italy, South Korea, Mexico, Poland, Portugal, Slovenia, and Spain), with the largest disparities (percentage points difference; p<0⋅05) in 2016: 13⋅8 (95% CI 12⋅27 to 15⋅33) in South Korea, 10⋅9 (8⋅94 to 12⋅86) in Spain, and 10⋅0 (8⋅04 to 11⋅96) in Italy. However, age differences were never significant in nine of 22 countries (Austria, Belgium, China, Denmark, France, the Netherlands, Sweden, Switzerland, and the USA), and in four of these countries (Belgium, Denmark, the Netherlands, and Sweden) the older-old reported less pain than the younger-old. Age disparities slightly increased over time in most countries, with the largest rises in Italy, Slovenia, and Spain (3⋅6 [0⋅93 to 6⋅27], 3⋅7 [–2⋅01 to 9⋅41], and 3⋅9 [1⋅33 to 6⋅47], respectively), while they declined in Belgium, Denmark, the Netherlands, and Sweden.

The overall disparity landscape for 2016, showing lower disparities in western and northern European countries (Austria, Denmark, France, Germany, and the Netherlands) and greater disparities in southern European countries (Greece, Italy, Portugal, and Spain) and South Korea, compared with countries in other regions is shown in the appendix (p 17). The intersectoral disparities for females with low levels of education aged older than 60 years shows the widest gaps in South Korea and the smallest in Denmark (appendix p 18).

## Discussion

The experience of pain is a major concern for individuals and societies, especially among older populations.^1–8^ This study documented international pain trends and disparities among adults aged 50 years and older in 22 countries. Nearly half of these adults reported pain, with prevalence generally increasing over time, although trends were stable in some countries and slightly decreased in China. Notably, pain prevalence increased by 12⋅1 percentage points, highlighting a burden needing urgent attention within the UN Decade of Healthy Ageing and global efforts on musculoskeletal pain.^13,14^ In 2016, Germany, Greece, and Italy had the largest population share of those aged 65 years and older; from 2000–24 China, Israel, and the Netherlands were ageing fastest, shaping pain projections (appendix p 16).

These findings correspond with previous studies indicating rising pain levels in the USA^17^ and mixed trends across Europe,^9^ and are largely consistent with recent Global Burden of Disease findings for low back pain.^7^ Similar to Zimmer and colleagues,^9^ we observed significant unadjusted increases in Belgium, Estonia, and Slovenia, whereas trends remained steady in Austria, Denmark, and Sweden. Notably, the flat trends in Sweden differ from slight decreases reported during 2011–13 for adults aged 65 years and older by Larsson and colleagues.^18^ Our findings of steady trends in Switzerland differ from the slight increases reported by Zimmer and colleagues, likely due to methodological differences (the latter study calculates separate trends for 2004–11 and 2013–15, whereas we focussed on 2006–16 trends adjusting for changes in the type of pain question). For other countries, our results aligned only partly with one of the two periods analysed by Zimmer and colleagues (indicating potential non-linear trends) or were not comparable as additional countries were included in our study (eg, China, England, Greece, Israel, Mexico, Portugal, South Korea, and the USA).

Notably, our study documented a decreasing prevalence of pain over time in China, a claim previously made without sufficient evidence in cross-sectional studies.^26^ Our finding aligns with previous research documenting historical decreases in low back pain in China, although this same research also noted increases in neck pain.^7,27^ The observed decreases in overall pain prevalence could be attributed to advances in health care and substantial automation of labour-intensive industries, which have reduced physical strain. Additionally, economic growth, improved living standards, and survivor bias—whereby healthier people tend to live longer than less heathy people—could also contribute to these trends. Given the stark contrast between China’s decline and the sharp increases observed elsewhere, further research is needed to identify the protective factors underlying stable or declining pain levels in certain countries.

The overall trends observed are unlikely to be fully explained by population feminisation, rising education levels, or population ageing, as these demographic shifts were minimal during the study period. Instead, country-specific factors likely drive historical variations in pain prevalence. The regional patterns observed in our study further suggest some super-national determinants: western and northern Europe generally had lower pain disparities, while southern Europe and parts of Asia, notably South Korea, had higher disparities. These regional patterns could reflect typological differences in health care and health insurance, where more comprehensive systems and coverage might contribute to lower pain disparities. However, substantial variability within regions highlights the role of each country’s specific configuration of policies and cultural practices on pain prevention and management.

Consistent with previous literature,^9–12,22,23^ we found overall higher pain prevalence among women,^7,20,24^ those with less education,^21^ and older individuals,^1,11^ highlighting well documented disparities. However, our study adds evidence showing that these disparities intersect and vary considerably across countries in three continents. For example, in 2016, Austria displayed the smallest pain disparities, while South Korea had the largest, with differences of up to 23 percentage points by sex, 28 points by education, and 14 points by age. Pain disparities slightly widened in some countries, such as age disparities in Spain, where austerity following the 2008 global financial crisis, weakening family support, and increasing health-care inequalities were more pronounced than elsewhere, reducing access to services for older adults and amplifying vulnerability to pain.^28^ Notably, educational and age disparities were never significant in the Netherlands, an unexpected and inspiring finding that could offer policy insights, possibly linked to its uniquely comprehensive disability benefits, strong workplace protections, and highly egalitarian education system.

Sex disparities in pain could be influenced by social factors, including differences in caregiving responsibilities, work conditions, pain treatment practices, and contraceptive use, as well as biological mechanisms such as variations in pain sensitivity and analgesic response, influenced by hormonal and genetic differences.^20^ Educational disparities likely reflect differences in health-care access and occupational conditions,^21,22^ whereas age disparities could stem from physical decline, lifetime injuries, lifestyles, and cumulative diseases, but could also be shaped by retirement policies, disability benefits, and pension systems.^11^ Moreover, psychosocial stressors—such as financial strain, food insecurity, childhood maltreatment, and loneliness—are increasingly recognised as pain risk factors.^29^ Disadvantaged groups, often more exposed and susceptible to these hardships, might experience exacerbated pain disparities depending on national contexts and social policies.^3,15^ Further research is needed to identify socially modifiable factors that could alleviate pain burden and reduce disparities, guiding more effective interventions.

Given population ageing, understanding international pain trends and disparities is crucial. By harmonising repeated individual-level measurements of any pain among older adults in three continents, this study moves pain research toward a more international perspective, isolating findings from methodological differences across single-country studies, and moving beyond condition specific estimates.^1,4–7,12,15,17,19^ The rapid rise in pain prevalence observed in most countries underscores the urgency of understanding why some nations have maintained stable or declining pain levels. The wide variations in pain prevalence and disparities further underscore the need for broad-scale prevention and intervention efforts targeted at specific countries and subgroups. However, these initiatives must proceed cautiously to avoid unintended consequences, as increased recognition of pain as a public health problem has previously led to the aggressive marketing of opioids for chronic pain—for which they were often ineffective or harmful, increasing the risk of addiction and overdose.^6^ Identifying country-specific disparities by sex, education, and age provides further insights for targeted interventions. Each country’s unique cultural and social context, along with its demographic composition shapes the prevalence, distribution, and reporting of pain over time. These findings align with influential frameworks on social determinants of health (eg, WHO’s model) that emphasise both structural (eg, the socioeconomic and political context) and individual-level drivers of health disparities (eg, sex).^13,14^ Overall, our study broadens the understanding of pain from an individual issue to a societal concern, reinforcing the importance of addressing upstream determinants in pain prevention and policy.^2,15^

Our study shows social disparities in pain, hinting—but not proving—at implications for addressing inequalities.^10–12,15,30^ Pain prevention, education, and treatment options are especially needed in countries such as Slovenia and Spain, where pain levels and disparities are high and rising. Further research is needed to understand the protective social, cultural, economic, and policy factors underlying low or declining pain levels and disparities in countries such as Switzerland, Austria, Denmark, and China; and to assess whether these conditions can be successfully emulated and adapted elsewhere to mitigate pain burden and disparities.

Despite our study’s strengths, harmonised data require trade-offs given differences in baselines, missingness and attrition, misclassification, question wording, statistical weights, and coverage of low-income and middle-income countries. Our self-reported pain measure, although widely used and reasonably accurate, does not capture location, duration, cause, or impact, and is subject to recall and cultural differences in reporting.^25^ Modelling question type directly mitigates instrument variation. Further research should incorporate more detailed pain measures, explore drivers of international and temporal variation, model non-linear trends and international differences in disparities, identify the causes and mechanisms underlying pain disparities, and develop targeted interventions. As with all observational studies, residual confounding could remain, and alternative analytical approaches could add insights.

To the best of our knowledge, this study is the first to longitudinally examine international trends and disparities in pain among middle-aged and older adults in Europe, Asia, and the Americas. Pain affects nearly half of this population, with prevalence rising over time in most countries. We observed wide variations in pain prevalence across countries, time, and population subgroups. Our analyses of pain trends and disparities across multiple countries and continents underscores the need for a population public health approach and broad-scale prevention and intervention efforts, emphasising the crucial role of social factors in shaping pain trends and disparities.

## Supplementary Material

1

## Figures and Tables

**Table:** Unadjusted prevalence of pain in 22 countries, overall and by sex, education, and age

	Baseline		All timepoints pooled	
	n (%)	95% CI	n (%)	95% CI

Overall	110 373 (44⋅92%)	44⋅62–45⋅21	687 369 (43⋅21%)	43⋅10–43⋅33
Female	60 535 (50⋅18%)	49⋅79–50⋅58	385 083 (48⋅08%)	47⋅93–48⋅24
Male	49 838 (38⋅52%)	38⋅09–38⋅95	302 286 (37⋅01%)	36⋅84–37⋅18
Less than high school completed	63 489 (49⋅38%)	48⋅99–49⋅77	311 990 (49⋅84%)	49⋅67–50⋅02
High school completed or more	46 884 (38⋅88%)	38⋅43–39⋅32	375 379 (37⋅70%)	37⋅55–37⋅86
Aged 50–60 years	41 509 (41⋅61%)	41⋅14–42⋅09	205 266 (39⋅67%)	39⋅46–39⋅89
Aged older than 60 years	68 864 (46⋅91%)	46⋅54–47⋅28	482 103 (44⋅72%)	44⋅58–44⋅86

Unweighted pooled data. For baseline years and country–specific descriptives see the appendix (p 3).

## Research in context

### Evidence before this study

On Sept 10, 2025, we searched PubMed for papers published between January, 2015, and July, 2025, using the terms “chronic pain”, “older adults”, “aging”, “international”, “cross-national”, and “longitudinal”, screened reference lists, and followed anonymous reviewers’ recommendations, including key studies most pertinent to understanding any pain or chronic pain among middle-aged and older adults (ie, those aged 50 years and older) across countries. Experiences and reports of pain trends among middle-aged and older adults vary over time differently across countries, with documented increases in the USA and parts of Europe, and stability or declines elsewhere, mostly in Asia. Few efforts have harmonised data for cross-country comparisons beyond condition-specific pain. Most existing work relies on single-country data, non-representative samples, limited age groups, or heterogeneous measures, limiting comparability across studies. Outside Europe, pain is rarely assessed in national health surveys or is limited to simplistic pain yesterday questions, and Global Burden of Disease studies focus on low back pain rather than any pain. To the best of our knowledge, no previous research has harmonised individual-level longitudinal data across multiple continents to examine pain prevalence trends and the evolution of social disparities by sex, education, and age.

### Added value of this study

To the best of our knowledge, this is the first longitudinal, international study of any pain trends and disparities among adults aged 50 years and older using harmonised nationally representative individual-level data from 22 countries across Europe, Asia, and the Americas. Adjusting for measurement differences, our study provides directly measured, internationally comparable estimates of pain prevalence and disparities by sex, education, and age, as well as their intersection over time. Overall, the experience of pain rose markedly among adults aged 50 years and older in most countries, declined in China, and disparities substantially varied across countries. Identifying countries with persistently low disparities (eg, Denmark) and others with widening gaps (eg, South Korea), this study highlights the role that social and policy contexts could play in shaping pain outcomes. By extending beyond single-nation or cross-sectional perspectives, this study can help inform comparative policy analysis and the design of targeted interventions.

### Implications of all the available evidence

Pain affects nearly half of all adults aged 50 years and older, and prevalence is rising in most countries, with disparities varying markedly across countries. These patterns are neither inevitable nor universal, pointing to opportunities for targeted prevention and management and equity-focused interventions. Countries maintaining low pain levels and disparities can inform policy transfer and system strengthening. Our findings underscore that pain is a barrier to WHO’s Healthy Ageing goals; coordinated action under the UN’s Decade of Healthy Ageing, including integration of pain prevention and management into broader population public health strategies for healthy longevity, remains critical. Policies must address structural drivers of pain and design socially responsive, equity-oriented interventions, especially as populations age. Initiatives should proceed cautiously to avoid unintended consequences, noting that past recognition of pain fuelled harmful opioid marketing and distribution. At the same time, research should refine pain measures, analyse non-linear variation, and uncover mechanisms to inform targeted interventions.
