# Supplementary material for: International trends and social disparities in pain of adults aged 50 years and older in 22 countries across Europe, Asia, and the Americas: a longitudinal population-based study
Source: Lancet Healthy Longev. Author manuscript; Available in PMC 2026 Jul 21. (PMC13387818; doi:10.1016/j.lanhl.2025.100808)
Supplement: 1 [file NIHMS2150701-supplement-1.pdf]

THE LANCET
Healthy Longevity

Supplementary appendix

This appendix formed part of the original submission and has been peer reviewed.
We post it as supplied by the authors.

Supplement to: Calvo E, Medina JT, Grol-Prokopczyk H, et al. International trends and social disparities in pain of adults aged 50 years and older in 22 countries across Europe, Asia, and the Americas: a longitudinal population-based study. *Lancet Healthy Longev* 2026.
https://doi.org/10.1016/j.lanhl.2025.100808

International trends and social disparities in pain: A longitudinal population-based study of adults aged 50 and older in 22 countries across Europe, Asia, and the Americas

SUPPLEMENTARY MATERIAL TABLE OF CONTENTS

Tables

Supplementary Table 1. Sample description, overall and by country 2

Supplementary Table 2. Descriptive statistic, by country 3

Supplementary Table 3. Longitudinal logistic regression results for model 1, overall estimates 4

Supplementary Table 4. Longitudinal logistic regression results for model 2, sex disparities 5

Supplementary Table 5. Longitudinal logistic regression results for model 3, educational disparities 6

Supplementary Table 6. Longitudinal logistic regression results for model 4, age disparities 7

Supplementary Table 7. Longitudinal logistic regression results for model 5, high-risk group disparities 8

Supplementary Table 8. Variance Inflation Factors (VIFs) 9

Supplementary Table 9. Longitudinal logistic regression results for model 2b, sex disparities using 3-level education 11

Supplementary Table 10. Longitudinal logistic regression results for model 3b, 3-level educational disparities 12

Supplementary Table 11. Longitudinal logistic regression results for model 4b, age disparities using 3-level education 13

Supplementary Table 12. Changes in pain prevalence by country, 2006-2016 14

Supplementary Table 13. Changes in social disparities in pain by country, 2006-2016 15

Supplementary Table 14. Sociodemographic and economic characteristics at 2016 by country 16

Figures

Supplementary Figure 1. Overall pain social disparity index across continents, 2016 17

Supplementary Figure 2. Pain prevalence by risk group in 22 countries, 2006-2016 18

References

Supplementary References 19

Supplementary Table 1. Sample description, overall and by country

Country	Survey	Age[2]	Interview method[3]	Years	Years span	N	Pain prevalence[1]		
							%	95% CI	
China	CHARLS	45+	CAPI	2011-2013-2015	4	18975	33·65	33·20	34·10
England	ELSA	50+	CAPI	2002-2004-2006-2008-2010-2012-2014-2016-2018	14	17051	39·58	39·23	39·92
United States	HRS	51+	CAPI, CATI	1998-2000-2002-2004-2006-2008-2010-2012-2014-2016-2018	18	37316	34·92	34·71	35·12
South Korea	KLOSA	45+	CAPI	2006-2008-2010-2012-2014-2016-2018	10	10808	63·24	62·83	63·64
Mexico	MHAS	50+	CAPI, PAPI	2001-2003-2012-2015	11	20166	39·43	39·00	39·85
Austria	SHARE	50+	CAPI	2004-2007-2011-2013-2015-2017	13	6192	44·83	44·06	45·61
Belgium				2005-2007-2011-2013-2015-2017	12	9500	49·87	49·24	50·49
Czech Republic				2007-2011-2013-2015-2017	10	8355	55·87	55·17	56·57
Denmark				2004-2007-2011-2013-2015-2017	13	5644	38·65	37·88	39·42
Estonia				2011-2013-2015	4	7622	56·64	55·92	57·37
France				2004-2006-2011-2013-2015-2017	13	7997	57·87	57·20	58·55
Germany				2004-2007-2011-2013-2015-2017	13	8529	45·26	44·53	45·99
Greece				2004-2007-2015-2017	13	6361	42·44	41·58	43·31
Israel				2006-2010-2013-2015	9	3768	47·24	46·22	48·26
Italy				2004-2007-2011-2013-2015-2017	13	8265	54·95	54·27	55·64
Netherlands				2004-2007-2011-2013	9	6347	37·20	36·35	38·05
Poland				2007-2011-2015	8	3013	59·42	58·27	60·57
Portugal				2011-2015	4	2137	57·47	55·84	59·07
Slovenia				2011-2013-2015	4	5227	54·88	53·89	55·86
Spain				2004-2007-2011-2013-2015-2015	11	8620	51·58	50·91	52·24
Sweden				2004-2007-2011-2013-2015-2016	12	6550	43·93	43·19	44·68
Switzerland				2004-2007-2011-2013-2015-2017	13	4461	35·88	35·05	36·73
Total					10·5	212904	43·21	43·10	43·33

Note: All surveys have refreshment samples and thus can be characterized as open cohorts.

[1] Prevalence of pain (unadjusted) for all time-points pooled.

[2] Age of eligibility

[3] CAPI=Computer Assisted Personal Interviewing; CATI=Computer Assisted Telephone Interviewing; PAPI=Pen And Paper Interviewing

Supplementary Table 2. Descriptive statistic, by country

	Pain prevalence	Baseline			Last follow-up wave			All time-points pooled		
**Country**	**Age standardized**	**Age (mean)**	**Educational attainment**	**Female (%)**	**Age (mean)**	**Educational attainment**	**Female (%)**	**Age (mean)**	**Educational attainment**	**Female (%)**
Austria	44·20	65·70	2·09	57·71	74.66	2·16	62·79	67·46	2·26	57·79
Belgium	49·90	64·98	1·92	53·09	73.04	2·18	56·70	66·28	2·16	54·57
China	33·59	62·70	0·64	50·43	62.88	0·72	51·04	62·76	0·69	50·85
Czech Republic	54·63	64·49	1·64	56·87	72.38	1·75	60·46	66·96	1·83	58·35
Denmark	38·91	64·86	2·35	53·39	71.19	2·71	56·60	65·82	2·60	53·83
England	38·69	65·35	1·91	53·06	68.70	2·45	55·51	66·99	2·20	54·21
Estonia	55·54	66·72	2·27	59·33	68.42	2·40	60·55	67·77	2·32	60·02
France	57·11	65·19	1·62	55·38	72.32	2·04	58·22	67·05	1·83	56·49
Germany	44·78	64·54	2·36	53·30	72.10	2·63	54·14	65·97	2·48	52·77
Greece	40·45	64·81	1·58	53·56	71.40	1·77	57·76	66·95	1·63	55·38
Israel	45·05	64·58	2·01	54·43	70.28	2·38	57·41	67·67	2·25	55·88
Italy	52·20	64·92	1·31	55·14	73.35	1·41	56·69	67·22	1·40	54·56
South Korea	59·61	65·21	1·24	56·45	69.88	1·58	57·65	67·01	1·43	56·95
Mexico	39·17	63·13	0·97	54·19	67.01	1·16	57·71	64·95	1·06	56·34
Netherlands	37·31	63·85	1·81	53·13	66.37	2·08	54·95	65·29	1·98	54·46
Poland	57·71	64·31	1·70	55·70	71.20	1·80	58·02	66·74	1·78	56·29
Portugal	56·28	65·31	1·35	56·03	67.67	1·34	54·85	66·40	1·34	55·49
Slovenia	53·48	65·70	1·94	56·08	67.68	1·98	56·78	66·91	1·97	56·52
Spain	48·25	66·91	1·07	57·64	74.58	1·13	58·03	68·99	1·16	54·93
Sweden	44·37	65·40	1·97	53·07	74.78	2·27	57·46	68·70	2·20	53·82
Switzerland	35·47	65·40	1·80	52·89	72.91	2·14	57·05	66·94	2·21	54·49
United States	35·10	67·22	1·90	57·06	68.06	2·38	58·47	68·22	2·16	57·96
Total	46·44	65·08	1·58	54·85	67.90	1·75	56·46	66·99	1·87	56·02

Note: Age-standardized prevalence of pain for all time-points pooled, estimated using two age groups (50-60 and >60 years), applying WHO 2000-2025 standard weights (45.4% and 54.7%, respectively). Educational attainment: 0=No education or primary uncompleted; 1=Primary completed but high school uncompleted; 2=High school completed; 3=Some college; 4=College completed or more.

Supplementary Table 3. Longitudinal logistic regression results for model 1, overall estimates

	Main term		Interaction with survey year	
	Coef.	[95% CI]	Coef.	[95% CI]
**Country**				
Austria	115·205***	[85·29,145·12]	-0·057***	[-0·07,-0·04]
Belgium	29·031**	[7·36,50·70]	-0·014**	[-0·03,-0·00]
China	218·997***	[186·07,251·93]	-0·109***	[-0·13,-0·09]
Czech Republic	38·498*	[5·88,71·12]	-0·019*	[-0·03,-0·00]
Denmark	117·357***	[91·40,143·32]	-0·059***	[-0·07,-0·05]
England	58·726***	[49·06,68·39]	-0·029***	[-0·03,-0·02]
Estonia	-147·939***	[-200·57,-95·31]	0·074***	[0·05,0·10]
France	-133·128***	[-155·15,-111·11]	0·067***	[0·06,0·08]
Germany	130·835***	[106·72,154·95]	-0·065***	[-0·08,-0·05]
Greece	33·394**	[11·51,55·28]	-0·017**	[-0·03,-0·01]
Israel	-0·176	[-36·53,36·18]	0·000	[-0·02,0·02]
Italy	-105·414***	[-127·24,-83·58]	0·053***	[0·04,0·06]
South Korea	93·668***	[80·75,106·58]	-0·046***	[-0·05,-0·04]
Mexico	135·935***	[126·15,145·72]	-0·068***	[-0·07,-0·06]
Netherlands	51·182**	[19·75,82·62]	-0·026**	[-0·04,-0·01]
Poland	-58·683**	[-94·23,-23·14]	0·030**	[0·01,0·05]
Portugal	-126·979**	[-217·98,-35·97]	0·063**	[0·02,0·11]
Slovenia	-257·481***	[-328·63,-186·33]	0·128***	[0·09,0·16]
Spain	-110·472***	[-133·49,-87·46]	0·055***	[0·04,0·07]
Sweden	108·514***	[85·76,131·27]	-0·054***	[-0·07,-0·04]
Switzerland	111·886***	[79·66,144·11]	-0·056***	[-0·07,-0·04]
**Survey year**	0·061***	[0·06,0·06]	-	-
**Site-specific pain question**	0·979***	[0·94,1·02]	-	-
**Constant**	-123·695***	[-128·24,-119·15]	-	-

Note: * p<0·05, ** p<0·01, *** p<0·001. The United States is the reference category for the country fixed effects. All coefficients are from the same regression model, which adjusts simultaneously for survey year and type of pain question. Columns display main effects and interaction terms from this single model.

Supplementary Table 4. Longitudinal logistic regression results for model 2, sex disparities

	Main term		Interaction with survey year		Interaction with sex	
	Coef.	[95% CI]	Coef.	[95% CI]	Coef.	[95% CI]
**Country**						
Austria	115·269***	[85·64,144·89]	-0·057***	[-0·07,-0·04]	-0·247***	[-0·39,-0·10]
Belgium	27·028*	[5·49,48·56]	-0·013*	[-0·02,-0·00]	0·074	[-0·05,0·19]
China	227·578***	[194·72,260·44]	-0·114***	[-0·13,-0·10]	0·377***	[0·28,0·47]
Czech Republic	33·970*	[1·55,66·39]	-0·017*	[-0·03,-0·00]	-0·049	[-0·18,0·08]
Denmark	118·439***	[92·73,144·14]	-0·059***	[-0·07,-0·05]	-0·138	[-0·29,0·01]
England	56·175***	[46·54,65·81]	-0·028***	[-0·03,-0·02]	-0·077	[-0·17,0·01]
Estonia	-152·574***	[-204·88,-100·26]	0·076***	[0·05,0·10]	-0·181**	[-0·31,-0·05]
France	-133·376***	[-155·26,-111·49]	0·067***	[0·06,0·08]	-0·141*	[-0·27,-0·01]
Germany	127·431***	[103·63,151·23]	-0·063***	[-0·08,-0·05]	-0·181**	[-0·31,-0·05]
Greece	41·122***	[19·13,63·11]	-0·021***	[-0·03,-0·01]	0·755***	[0·60,0·91]
Israel	3·647	[-32·58,39·88]	-0·002	[-0·02,0·02]	0·135	[-0·04,0·31]
Italy	-103·125***	[-124·96,-81·29]	0·051***	[0·04,0·06]	0·545***	[0·42,0·67]
South Korea	94·317***	[81·20,107·43]	-0·047***	[-0·05,-0·04]	0·982***	[0·88,1·09]
Mexico	141·443***	[131·71,151·18]	-0·071***	[-0·08,-0·07]	0·368***	[0·28,0·46]
Netherlands	48·272**	[17·11,79·43]	-0·024**	[-0·04,-0·01]	0·145	[-0·01,0·30]
Poland	-56·628**	[-92·06,-21·19]	0·028**	[0·01,0·05]	0·076	[-0·13,0·28]
Portugal	-116·826*	[-208·66,-24·99]	0·058*	[0·01,0·10]	0·840***	[0·59,1·09]
Slovenia	-247·191***	[-317·97,-176·41]	0·123***	[0·09,0·16]	-0·082	[-0·25,0·08]
Spain	-107·462***	[-130·51,-84·41]	0·053***	[0·04,0·06]	0·702***	[0·58,0·83]
Sweden	102·581***	[80·00,125·17]	-0·051***	[-0·06,-0·04]	0·283***	[0·14,0·42]
Switzerland	103·167***	[71·30,135·03]	-0·052***	[-0·07,-0·04]	-0·168*	[-0·33,-0·00]
**Survey year**	0·057***	[0·05,0·06]	-	-	-	-
**Site-specific pain question**	0·974***	[0·93,1·01]	-	-	-	-
**Female**	-8·189**	[-14·30,-2·08]	0·004**	[0·00,0·01]	-	-
**High school completed or more**	-0·788***	[-0·81,-0·76]	-	-	-	-
**More than 60 years old**	0·198***	[0·18,0·22]	-	-	-	-
**Constant**	-115·374***	[-121·23,-109·52]	-	-	-	-

Note: * p<0·05, ** p<0·01, *** p<0·001. The United States is the reference category for the country fixed effects. All coefficients are from the same regression model, which adjusts simultaneously for all covariates. Columns display main effects and interaction terms from this single model.

Supplementary Table 5. Longitudinal logistic regression results for model 3, educational disparities

	Main term		Interaction with survey year		Interaction with education	
	Coef.	[95% CI]	Coef.	[95% CI]	Coef.	[95% CI]
**Country**						
Austria	116·462***	[86·80,146·12]	-0·058***	[-0·07,-0·04]	0·095	[-0·07,0·26]
Belgium	29·776**	[8·17,51·38]	-0·015**	[-0·03,-0·00]	0·196**	[0·07,0·32]
China	220·555***	[187·45,253·66]	-0·110***	[-0·13,-0·09]	-0·355***	[-0·50,-0·21]
Czech Republic	33·708*	[1·16,66·25]	-0·016*	[-0·03,-0·00]	-0·101	[-0·23,0·03]
Denmark	121·903***	[96·18,147·63]	-0·061***	[-0·07,-0·05]	0·317***	[0·14,0·50]
England	53·667***	[43·93,63·41]	-0·027***	[-0·03,-0·02]	-0·199***	[-0·29,-0·10]
Estonia	-149·725***	[-202·08,-97·37]	0·075***	[0·05,0·10]	-0·046	[-0·19,0·10]
France	-129·698***	[-151·66,-107·74]	0·065***	[0·05,0·08]	0·279***	[0·15,0·41]
Germany	129·932***	[106·09,153·78]	-0·065***	[-0·08,-0·05]	0·069	[-0·11,0·25]
Greece	32·223**	[10·17,54·28]	-0·016**	[-0·03,-0·01]	-0·497***	[-0·65,-0·34]
Israel	1·566	[-34·74,37·87]	-0·001	[-0·02,0·02]	-0·191*	[-0·38,-0·01]
Italy	-107·956***	[-130·01,-85·90]	0·054***	[0·04,0·06]	-0·372***	[-0·51,-0·23]
South Korea	78·045***	[64·67,91·42]	-0·038***	[-0·04,-0·03]	-1·258***	[-1·37,-1·15]
Mexico	134·127***	[123·46,144·80]	-0·067***	[-0·07,-0·06]	-0·332***	[-0·46,-0·20]
Netherlands	54·955***	[23·73,86·18]	-0·028***	[-0·04,-0·01]	0·511***	[0·36,0·66]
Poland	-56·990**	[-92·53,-21·45]	0·029**	[0·01,0·05]	-0·051	[-0·26,0·16]
Portugal	-119·873*	[-211·25,-28·50]	0·060**	[0·01,0·11]	-0·507**	[-0·81,-0·20]
Slovenia	-246·743***	[-317·64,-175·85]	0·123***	[0·09,0·16]	-0·209*	[-0·38,-0·04]
Spain	-112·804***	[-136·15,-89·46]	0·056***	[0·04,0·07]	-0·478***	[-0·63,-0·32]
Sweden	106·852***	[84·15,129·56]	-0·053***	[-0·06,-0·04]	0·250***	[0·11,0·39]
Switzerland	111·228***	[79·10,143·36]	-0·056***	[-0·07,-0·04]	0·283**	[0·09,0·47]
**Survey year**	0·057***	[0·05,0·06]	-	-	-	-
**Site-specific pain question**	0·972***	[0·93,1·01]	-	-	-	-
**Female**	0·692***	[0·67,0·71]	-	-	-	-
**High school completed or more**	-8·890*	[-15·74,-2·04]	0·004*	[0·00,0·01]	-	-
**More than 60 years old**	0·181***	[0·16,0·20]	-	-	-	-
**Constant**	-114·655***	[-121·34,-107·97]	-	-	-	-

Note: * p<0·05, ** p<0·01, *** p<0·001. The United States is the reference category for the country fixed effects. All coefficients are from the same regression model, which adjusts simultaneously for all covariates. Columns display main effects and interaction terms from this single model.

Supplementary Table 6. Longitudinal logistic regression results for model 4, age disparities

	Main term		Interaction with survey year		Interaction with age	
	Coef.	[95% CI]	Coef.	[95% CI]	Coef.	[95% CI]
**Country**						
Austria	119·055***	[88·85,149·26]	-0·059***	[-0·07,-0·04]	0·094	[-0·04,0·23]
Belgium	16·709	[-5·39,38·80]	-0·008	[-0·02,0·00]	-0·132*	[-0·23,-0·03]
China	222·570***	[189·49,255·65]	-0·111***	[-0·13,-0·09]	0·035	[-0·05,0·12]
Czech Republic	54·284**	[20·85,87·72]	-0·027**	[-0·04,-0·01]	0·340***	[0·22,0·46]
Denmark	104·915***	[78·34,131·49]	-0·052***	[-0·07,-0·04]	-0·175**	[-0·30,-0·05]
England	61·130***	[51·01,71·24]	-0·030***	[-0·04,-0·03]	0·156***	[0·09,0·22]
Estonia	-145·587***	[-198·17,-93·00]	0·073***	[0·05,0·10]	0·147*	[0·02,0·27]
France	-137·653***	[-160·23,-115·08]	0·069***	[0·06,0·08]	-0·01	[-0·12,0·10]
Germany	132·037***	[107·85,156·22]	-0·066***	[-0·08,-0·05]	0·161**	[0·04,0·28]
Greece	66·484***	[43·34,89·63]	-0·033***	[-0·04,-0·02]	0·578***	[0·44,0·72]
Israel	34·265	[-3·74,72·27]	-0·017	[-0·04,0·00]	0·523***	[0·36,0·69]
Italy	-78·381***	[-100·79,-55·97]	0·039***	[0·03,0·05]	0·563***	[0·45,0·68]
South Korea	141·146***	[127·39,154·90]	-0·070***	[-0·08,-0·06]	0·878***	[0·80,0·96]
Mexico	143·618***	[133·38,153·85]	-0·072***	[-0·08,-0·07]	0·184***	[0·11,0·25]
Netherlands	34·308*	[2·35,66·27]	-0·017*	[-0·03,-0·00]	-0·129	[-0·27,0·01]
Poland	-43·201*	[-81·10,-5·31]	0·022*	[0·00,0·04]	0·256**	[0·08,0·43]
Portugal	-99·843*	[-191·99,-7·70]	0·050*	[0·00,0·10]	0·384**	[0·13,0·64]
Slovenia	-225·281***	[-296·92,-153·65]	0·112***	[0·08,0·15]	0·387***	[0·22,0·55]
Spain	-77·076***	[-100·64,-53·51]	0·038***	[0·03,0·05]	0·667***	[0·55,0·79]
Sweden	94·615***	[70·91,118·32]	-0·047***	[-0·06,-0·04]	-0·092	[-0·23,0·04]
Switzerland	109·646***	[76·77,142·52]	-0·055***	[-0·07,-0·04]	0·153*	[0·00,0·30]
**Survey year**	0·051***	[0·05,0·06]	-	-	-	-
**Site-specific pain question**	0·975***	[0·93,1·01]	-	-	-	-
**Female**	0·709***	[0·69,0·73]	-	-	-	-
**High school completed or more**	-0·777***	[-0·80,-0·75]	-	-	-	-
**More than 60 years old**	-28·994***	[-36·33,-21·66]	0·014***	[0·01,0·02]	-	-
**Constant**	-104·104***	[-111·31,-96·90]	-	-	-	-

Note: * p<0·05, ** p<0·01, *** p<0·001. The United States is the reference category for the country fixed effects. All coefficients are from the same regression model, which adjusts simultaneously for all covariates. Columns display main effects and interaction terms from this single model.

Supplementary Table 7. Longitudinal logistic regression results for model 5, high-risk group disparities

	Main term		Interaction with survey year		Interaction with intersect	
	Coef.	[95% CI]	Coef.	[95% CI]	Coef.	[95% CI]
**Country**						
Austria	114·773***	[85·15,144·40]	-0·057***	[-0·07,-0·04]	0·217*	[0·03,0·40]
Belgium	30·191**	[8·68,51·70]	-0·015**	[-0·03,-0·00]	0·144	[-0·00,0·29]
China	238·964***	[206·08,271·85]	-0·119***	[-0·14,-0·10]	0·224***	[0·12,0·33]
Czech Republic	47·363**	[14·90,79·82]	-0·023**	[-0·04,-0·01]	0·382***	[0·23,0·54]
Denmark	113·916***	[88·23,139·60]	-0·057***	[-0·07,-0·04]	-0·147	[-0·37,0·08]
England	59·261***	[49·66,68·87]	-0·029***	[-0·03,-0·02]	0·219***	[0·11,0·33]
Estonia	-148·721***	[-200·96,-96·48]	0·074***	[0·05,0·10]	0·300**	[0·12,0·48]
France	-129·935***	[-151·78,-108·09]	0·065***	[0·05,0·08]	0·078	[-0·07,0·23]
Germany	124·786***	[100·97,148·60]	-0·062***	[-0·07,-0·05]	0·315**	[0·10,0·52]
Greece	50·090***	[27·99,72·19]	-0·025***	[-0·04,-0·01]	1·125***	[0·96,1·29]
Israel	12·084	[-24·22,48·39]	-0·006	[-0·02,0·01]	0·569***	[0·33,0·81]
Italy	-85·788***	[-107·77,-63·80]	0·043***	[0·03,0·05]	0·881***	[0·74,1·02]
Korea	119·031***	[105·80,132·27]	-0·059***	[-0·07,-0·05]	1·389***	[1·27,1·50]
Mexico	148·148***	[138·11,158·19]	-0·074***	[-0·08,-0·07]	0·051	[-0·04,0·14]
Netherlands	53·368***	[22·24,84·49]	-0·027***	[-0·04,-0·01]	-0·023	[-0·20,0·15]
Poland	-51·826**	[-87·29,-16·37]	0·026**	[0·01,0·04]	0·529***	[0·29,0·77]
Portugal	-90·434	[-182·39,1·53]	0·045	[-0·00,0·09]	0·987***	[0·71,1·26]
Slovenia	-243·676***	[-314·48,-172·88]	0·121***	[0·09,0·16]	0·719***	[0·51,0·93]
Spain	-86·291***	[-109·55,-63·03]	0·043***	[0·03,0·05]	0·918***	[0·79,1·05]
Sweden	106·882***	[84·35,129·42]	-0·053***	[-0·06,-0·04]	0·075	[-0·09,0·25]
Switzerland	110·808***	[78·90,142·72]	-0·055***	[-0·07,-0·04]	0·000	[-0·22,0·22]
**Survey year**	0·060***	[0·06,0·06]	-	-	-	-
**Intersect (high risk pain group)**	7·961*	[0·02,15·91]	-0·004	[-0·01,0·00]	-	-
**Site-specific pain question**	0·967***	[0·93,1·01]	-	-	-	-
**Constant**	-122·183***	[-126·81,-117·56]	-	-	-	-

* p<0.05, ** p<0.01, *** p<0.001 The United States is the reference category for the country fixed effects. All coefficients are from the same regression model, which adjusts simultaneously for all covariates. Columns display main effects and interaction terms from this single model.

Supplementary Table 8. Variance Inflation Factors (VIFs)

Variable	Model 1	Model 2 * with sex	Model 3 * with education	Model 4 * with age	Model 5 * with intersect
**Mean**	2.21	2.74	3.05	3.55	2.15
**Country**					
Austria	1.67	3.13	4.59	4.61	1.88
Belgium	1.74	3.08	3.32	3.85	1.99
China	4.48	5.90	5.22	6.25	4.82
CzechRepublic	2.37	3.86	3.74	5.05	2.63
Denmark	1.50	2.76	5.59	3.48	1.65
England	1.26	2.79	3.73	4.44	1.50
Estonia	5.21	6.79	7.67	7.93	5.42
France	1.55	2.96	2.98	3.96	1.86
Germany	1.40	2.63	7.76	3.69	1.52
Greece	1.29	2.60	2.19	3.79	1.67
Israel	1.30	2.61	3.07	4.07	1.48
Italy	1.60	2.95	2.26	4.45	2.04
Korea	4.10	5.73	5.38	6.81	4.63
Mexico	1.28	2.93	2.12	3.77	1.80
Netherlands	1.32	2.59	2.50	3.44	1.62
Poland	1.50	2.82	2.88	3.87	1.80
Portugal	3.35	4.67	3.67	5.39	3.76
Slovenia	5.58	6.93	7.61	7.52	5.80
Spain	1.70	3.10	2.23	4.85	2.26
Sweden	1.39	2.64	2.90	5.40	1.63
Switzerland	1.62	2.88	4.51	3.98	1.81
**Interaction country - survey year**					
Austria	1.41	1.41	1.41	1.42	1.41
Belgium	1.44	1.44	1.46	1.45	1.44
China	4.39	4.39	4.45	4.40	4.39
Czech Republic	2.03	2.03	2.05	2.09	2.03
Denmark	1.37	1.37	1.37	1.39	1.37
England	1.25	1.26	1.29	1.28	1.26
Estonia	4.68	4.68	4.69	4.68	4.68
France	1.27	1.27	1.28	1.29	1.27
Germany	1.31	1.31	1.32	1.32	1.32
Greece	1.24	1.24	1.26	1.29	1.25
Israel	1.19	1.19	1.20	1.23	1.19
Italy	1.41	1.41	1.44	1.42	1.41

Korea	1.32	1.32	1.38	1.33	1.34
Mexico	1.34	1.34	1.59	1.37	1.38
Netherlands	1.16	1.16	1.17	1.18	1.16
Poland	1.33	1.34	1.35	1.43	1.33
Portugal	3.18	3.18	3.19	3.22	3.19
Slovenia	5.37	5.37	5.38	5.43	5.37
Spain	1.51	1.52	1.56	1.53	1.53
Sweden	1.27	1.27	1.30	1.34	1.27
Switzerland	1.41	1.41	1.45	1.44	1.41
**Survey year**	2.28	3.86	5.46	5.20	2.40
**Site-specific pain question**	7.07	7.07	7.08	7.08	7.07
**Female**	-	3.55	1.02	1.01	-
**High school completed or more**	-	1.33	4.60	1.33	-
**More than 60 years old**	-	1.05	1.06	3.66	-
**Intersect (high risk pain group)**	-	-	-	-	5.50
**Interaction: * - survey year**	-	2.65	3.36	4.04	1.56
**Interaction: country - ***					
Austria	-	2.55	4.10	4.19	1.33
Belgium	-	2.46	2.69	3.33	1.47
China	-	2.53	1.30	2.96	2.05
Czech Republic	-	2.63	2.57	4.33	1.49
Denmark	-	2.33	5.32	3.20	1.21
England	-	2.86	3.64	4.55	1.85
Estonia	-	2.74	3.83	4.06	1.33
France	-	2.51	2.48	3.61	1.54
Germany	-	2.30	7.61	3.48	1.18
Greece	-	2.37	1.90	3.75	1.56
Israel	-	2.36	2.86	4.05	1.25
Italy	-	2.42	1.56	4.12	1.78
Korea	-	2.93	2.26	4.12	2.48
Mexico	-	2.74	1.28	3.40	2.20
Netherlands	-	2.31	2.13	3.10	1.42
Poland	-	2.37	2.49	3.92	1.39
Portugal	-	2.29	1.27	3.50	1.54
Slovenia	-	2.43	3.12	3.59	1.35
Spain	-	2.46	1.35	4.48	2.00
Sweden	-	2.34	2.61	5.45	1.41
Switzerland	-	2.34	4.31	3.68	1.26

Supplementary Table 9. Longitudinal logistic regression results for model 2b, sex disparities using 3-level education

	Main term		Interaction with survey year		Interaction with sex	
	Coef.	[95% CI]	Coef.	[95% CI]	Coef.	[95% CI]
Austria	115.564***	[85.95,145.18]	-0.057***	[-0.07,-0.04]	-0.233**	[-0.38,-0.09]
Belgium	26.727*	[5.20,48.26]	-0.013*	[-0.02,-0.00]	0.079	[-0.04,0.20]
China	229.548***	[196.70,262.40]	-0.115***	[-0.13,-0.10]	0.391***	[0.30,0.49]
Czech Republic	37.720*	[5.32,70.12]	-0.018*	[-0.03,-0.00]	-0.041	[-0.17,0.09]
Denmark	117.670***	[91.97,143.37]	-0.059***	[-0.07,-0.05]	-0.09	[-0.24,0.06]
England	56.663***	[47.03,66.30]	-0.028***	[-0.03,-0.02]	-0.091*	[-0.18,-0.00]
Estonia	-151.694***	[-204.03,-99.36]	0.076***	[0.05,0.10]	-0.150*	[-0.28,-0.02]
France	-131.058***	[-152.94,-109.18]	0.066***	[0.05,0.08]	-0.118	[-0.25,0.01]
Germany	127.395***	[103.59,151.20]	-0.063***	[-0.08,-0.05]	-0.190**	[-0.32,-0.06]
Greece	42.731***	[20.75,64.71]	-0.022***	[-0.03,-0.01]	0.755***	[0.60,0.91]
Israel	1.739	[-34.47,37.95]	-0.001	[-0.02,0.02]	0.136	[-0.04,0.31]
Italy	-101.367***	[-123.19,-79.55]	0.050***	[0.04,0.06]	0.562***	[0.43,0.69]
Korea	96.614***	[83.50,109.72]	-0.048***	[-0.05,-0.04]	0.987***	[0.88,1.09]
Mexico	142.759***	[133.02,152.50]	-0.071***	[-0.08,-0.07]	0.379***	[0.29,0.47]
Netherlands	48.352**	[17.19,79.51]	-0.025**	[-0.04,-0.01]	0.148	[-0.00,0.30]
Poland	-53.746**	[-89.16,-18.33]	0.027**	[0.01,0.04]	0.089	[-0.11,0.29]
Portugal	-113.999*	[-205.81,-22.19]	0.057*	[0.01,0.10]	0.855***	[0.60,1.11]
Slovenia	-247.731***	[-318.50,-176.96]	0.123***	[0.09,0.16]	-0.053	[-0.22,0.11]
Spain	-106.270***	[-129.32,-83.22]	0.053***	[0.04,0.06]	0.714***	[0.59,0.84]
Sweden	103.321***	[80.73,125.91]	-0.052***	[-0.06,-0.04]	0.306***	[0.17,0.45]
Switzerland	106.204***	[74.33,138.08]	-0.053***	[-0.07,-0.04]	-0.184*	[-0.35,-0.02]
Survey year	0.058***	[0.05,0.06]	-	-	-	-
Site-specific pain question	0.974***	[0.93,1.01]	-	-	-	-
Female	-8.385**	[-14.49,-2.28]	0.004**	[0.00,0.01]	-	-
High school completed	-0.613***	[-0.64,-0.58]	-	-	-	-
Some college or more	-0.966***	[-1.00,-0.94]	-	-	-	-
More than 60 years old	0.194***	[0.18,0.21]	-	-	-	-
Constant	-117.079***	[-122.94,-111.22]	-	-	-	-

Note: * p<0·05, ** p<0·01, *** p<0·001. The United States is the reference category for the country fixed effects. All coefficients are from the same regression model, which adjusts simultaneously for all covariates. Columns display main effects and interaction terms from this single model.

Supplementary Table 10. Longitudinal logistic regression results for model 3b, 3-level educational disparities

	Main term		Interaction with survey year		Interaction with High school completed		Interaction with: Some college or more	
	Coef.	[95% CI]	Coef.	[95% CI]	Coef.	[95% CI]	Coef.	[95% CI]
Austria	115.750***	[86.07,145.43]	-0.058***	[-0.07,-0.04]	0.017	[-0.16,0.19]	0.013	[-0.18,0.20]
Belgium	29.144**	[7.54,50.75]	-0.014**	[-0.03,-0.00]	0.155*	[0.01,0.30]	0.189**	[0.05,0.33]
China	221.441***	[188.35,254.54]	-0.110***	[-0.13,-0.09]	-0.328***	[-0.50,-0.16]	-0.672***	[-0.90,-0.44]
Czech Republic	34.643*	[2.08,67.20]	-0.017*	[-0.03,-0.00]	-0.229**	[-0.37,-0.09]	-0.179	[-0.36,0.01]
Denmark	120.161***	[94.42,145.90]	-0.060***	[-0.07,-0.05]	0.315**	[0.12,0.51]	0.242*	[0.04,0.44]
England	54.654***	[44.91,64.40]	-0.027***	[-0.03,-0.02]	-0.164**	[-0.28,-0.04]	-0.192***	[-0.30,-0.09]
Estonia	-149.604***	[-202.00,-97.21]	0.075***	[0.05,0.10]	0.061	[-0.11,0.23]	-0.176*	[-0.34,-0.02]
France	-128.685***	[-150.66,-106.71]	0.064***	[0.05,0.08]	0.241**	[0.09,0.39]	0.139	[-0.03,0.31]
Germany	128.195***	[104.29,152.10]	-0.064***	[-0.08,-0.05]	0.056	[-0.13,0.24]	-0.142	[-0.34,0.06]
Greece	32.775**	[10.72,54.83]	-0.016**	[-0.03,-0.01]	-0.569***	[-0.76,-0.38]	-0.521***	[-0.72,-0.32]
Israel	5.417	[-30.93,41.76]	-0.003	[-0.02,0.02]	-0.596***	[-0.83,-0.36]	0.078	[-0.13,0.28]
Italy	-107.590***	[-129.65,-85.53]	0.054***	[0.04,0.06]	-0.473***	[-0.64,-0.31]	-0.425***	[-0.63,-0.22]
Korea	78.261***	[64.83,91.70]	-0.038***	[-0.05,-0.03]	-1.244***	[-1.37,-1.12]	-1.649***	[-1.81,-1.49]
Mexico	134.586***	[123.92,145.25]	-0.067***	[-0.07,-0.06]	-0.334**	[-0.57,-0.10]	-0.262***	[-0.41,-0.12]
Netherlands	54.030***	[22.80,85.26]	-0.027***	[-0.04,-0.01]	0.494***	[0.31,0.68]	0.456***	[0.27,0.64]
Poland	-56.333**	[-91.90,-20.77]	0.028**	[0.01,0.05]	-0.166	[-0.39,0.06]	-0.200	[-0.51,0.11]
Portugal	-118.808*	[-210.13,-27.49]	0.059*	[0.01,0.10]	-0.585**	[-1.01,-0.16]	-0.477*	[-0.87,-0.08]
Slovenia	-251.216***	[-322.24,-180.20]	0.125***	[0.09,0.16]	-0.192*	[-0.38,-0.00]	-0.608***	[-0.84,-0.38]
Spain	-112.790***	[-136.13,-89.45]	0.056***	[0.04,0.07]	-0.448***	[-0.66,-0.23]	-0.537***	[-0.74,-0.34]
Sweden	107.693***	[84.99,130.40]	-0.054***	[-0.06,-0.04]	0.333***	[0.15,0.52]	0.209*	[0.05,0.37]
Switzerland	112.271***	[80.14,144.40]	-0.056***	[-0.07,-0.04]	0.111	[-0.10,0.32]	0.368***	[0.15,0.59]
Survey year	0.057***	[0.05,0.06]	-	-	-	-	-	-
Site-specific pain question	0.972***	[0.93,1.01]	-	-	-	-	-	-
Female	0.681***	[0.66,0.70]	-	-	-	-	-	-
High school completed	-8.656*	[-16.80,-0.51]	0.004*	[0.00,0.01]	-	-	-	-
Some college or more	-11.747**	[-19.59,-3.91]	0.005**	[0.00,0.01]	-	-	-	-
More than 60 years old	0.177***	[0.16,0.20]	-	-	-	-	-	-
Constant	-115.084***	[-121.78,-108.39]	-	-	-	-	-	-

Note: * p<0·05, ** p<0·01, *** p<0·001. The United States is the reference category for the country fixed effects. All coefficients are from the same regression model, which adjusts simultaneously for all covariates. Columns display main effects and interaction terms from this single model.

Supplementary Table 11. Longitudinal logistic regression results for model 4b, age disparities using 3-level education

	Main term		Interact with survey year		Interact with age	
	Coef.	[95% CI]	Coef.	[95% CI]	Coef.	[95% CI]
Austria	119.955***	[89.76,150.15]	-0.060***	[-0.07,-0.04]	0.115	[-0.02,0.25]
Belgium	16.811	[-5.28,38.90]	-0.008	[-0.02,0.00]	-0.118*	[-0.22,-0.02]
China	226.253***	[193.19,259.31]	-0.113***	[-0.13,-0.10]	0.070	[-0.01,0.15]
Czech Republic	59.359***	[25.94,92.78]	-0.029***	[-0.05,-0.01]	0.367***	[0.24,0.49]
Denmark	103.427***	[76.86,129.99]	-0.052***	[-0.06,-0.04]	-0.180**	[-0.31,-0.05]
England	61.857***	[51.74,71.97]	-0.031***	[-0.04,-0.03]	0.166***	[0.10,0.23]
Estonia	-144.272***	[-196.87,-91.67]	0.072***	[0.05,0.10]	0.165*	[0.04,0.29]
France	-133.944***	[-156.51,-111.38]	0.067***	[0.06,0.08]	0.021	[-0.09,0.13]
Germany	132.268***	[108.09,156.45]	-0.066***	[-0.08,-0.05]	0.171**	[0.05,0.29]
Greece	68.834***	[45.70,91.96]	-0.035***	[-0.05,-0.02]	0.597***	[0.46,0.74]
Israel	32.357	[-5.62,70.33]	-0.016	[-0.04,0.00]	0.533***	[0.37,0.70]
Italy	-75.574***	[-97.97,-53.18]	0.037***	[0.03,0.05]	0.591***	[0.48,0.71]
Korea	145.149***	[131.40,158.90]	-0.072***	[-0.08,-0.07]	0.916***	[0.84,1.00]
Mexico	145.329***	[135.10,155.56]	-0.073***	[-0.08,-0.07]	0.197***	[0.13,0.27]
Netherlands	34.962*	[3.01,66.91]	-0.018*	[-0.03,-0.00]	-0.114	[-0.25,0.02]
Poland	-37.397	[-75.27,0.47]	0.019	[-0.00,0.04]	0.302***	[0.12,0.48]
Portugal	-96.082*	[-188.19,-3.97]	0.048*	[0.00,0.09]	0.407**	[0.15,0.66]
Slovenia	-224.854***	[-296.46,-153.25]	0.112***	[0.08,0.15]	0.412***	[0.25,0.58]
Spain	-75.116***	[-98.67,-51.56]	0.037***	[0.03,0.05]	0.690***	[0.57,0.81]
Sweden	95.625***	[71.93,119.32]	-0.048***	[-0.06,-0.04]	-0.079	[-0.22,0.06]
Switzerland	113.452***	[80.57,146.33]	-0.057***	[-0.07,-0.04]	0.170*	[0.02,0.32]
Survey year	0.053***	[0.05,0.06]	-	-	-	-
Site-specific pain question	0.974***	[0.93,1.01]	-	-	-	-
Female	0.701***	[0.68,0.72]	-	-	-	-
High school completed	-0.594***	[-0.62,-0.56]	-	-	-	-
Some college or more	-0.961***	[-0.99,-0.93]	-	-	-	-
More than 60 years old	-28.316***	[-35.65,-20.99]	0.014***	[0.01,0.02]	-	-
Constant	-106.880***	[-114.09,-99.67]	-	-	-	-

Note: * p<0·05, ** p<0·01, *** p<0·001. The United States is the reference category for the country fixed effects. All coefficients are from the same regression model, which adjusts simultaneously for all covariates. Columns display main effects and interaction terms from this single model.

Supplementary Table 12. Changes in pain prevalence by country, 2006-2016

	2006			2016		
Country	%	95% CI		%	95% CI	
Austria	37.2	35.6	38.8	37.8	36.4	39.1
Belgium	39.3	38.0	40.6	46.2	45.1	47.3
China	38.2	36.5	40.0	31.6	30.7	32.4
Czech Republic	45.3	43.5	47.1	51.6	50.4	52.9
Denmark	32.5	31.1	34.0	32.9	31.6	34.1
England	38.5	37.9	39.1	43.1	42.4	43.9
Estonia	37.2	34.4	40.0	57.2	55.8	58.7
France	40.3	39.0	41.5	59.2	58.1	60.4
Germany	39.2	37.9	40.5	38.7	37.5	39.9
Greece	32.3	31.0	33.6	38.5	37.2	39.7
Israel	35.3	33.5	37.1	44.2	42.3	46.0
Italy	37.7	36.4	39.0	54.5	53.4	55.7
South Korea	46.4	45.4	47.5	48.7	47.6	49.7
Mexico	39.7	39.1	40.2	38.7	38.0	39.5
Netherlands	26.7	25.5	27.9	31.2	29.6	32.9
Poland	42.3	40.2	44.5	55.8	54.0	57.7
Portugal	36.7	31.9	41.5	55.2	52.4	57.9
Slovenia	30.6	26.9	34.2	58.1	56.3	59.8
Spain	34.4	33.2	35.7	51.5	50.4	52.6
Sweden	37.1	35.8	38.4	38.1	36.9	39.3
Switzerland	29.4	27.8	30.9	30.0	28.6	31.4
United States	33.6	33.3	34.0	42.4	41.9	42.8

Notes: Predicted prevalence estimates and 95% CIs based on model 1 (see Supplementary Table 3).

Supplementary Table 13. Changes in social disparities in pain by country, 2006-2016

	Sex						Education						Age					
	2006			2016			2006			2016			2006			2016		
Country	Pts.	95% CI		Pts.	95% CI		Pts.	95% CI		Pts.	95% CI		Pts.	95% CI		Pts.	95% CI	
Austria	3.8	1.2	6.4	4.4	2.0	6.8	8.2	5.4	11.0	7.7	5.1	10.3	0.9	-1.5	3.3	2.8	0.7	4.9
Belgium	8.4	6.3	10.5	9.5	7.6	11.4	6.9	4.8	9.0	6.6	4.6	8.6	-2.1	-4.0	-0.2	-0.1	-1.9	1.7
China	11.9	9.4	14.4	10.8	9.5	12.1	13.5	10.9	16.1	11.4	9.8	13.0	0.1	-2.1	2.3	1.6	0.5	2.7
Czech Republic	6.9	4.1	9.7	7.8	5.6	10.0	11.6	8.7	14.5	11.3	9.1	13.5	4.4	1.6	7.2	6.8	4.4	9.2
Denmark	5.1	2.6	7.6	5.7	3.5	7.9	4.8	2.1	7.5	4.2	1.6	6.8	-2.4	-4.5	-0.3	-0.7	-2.7	1.3
England	6.2	5.1	7.3	7.1	5.7	8.5	12.4	11.1	13.7	12.4	11.0	13.8	1.7	0.7	2.7	3.9	2.6	5.2
Estonia	4.7	0.7	8.7	5.8	3.3	8.3	10.2	6.2	14.2	10.4	7.7	13.1	1.6	-2.0	5.2	4.1	1.4	6.8
France	5.3	3.1	7.5	6.3	4.2	8.4	5.8	3.7	7.9	5.4	3.3	7.5	-0.4	-2.3	1.5	1.7	-0.5	3.9
Germany	4.9	2.7	7.1	5.5	3.3	7.7	8.9	6.0	11.8	8.2	5.4	11.0	1.8	-0.3	3.9	3.8	1.8	5.8
Greece	15.7	13.6	17.8	17.7	15.6	19.8	14.6	12.5	16.7	15.6	13.5	17.7	6.0	4.2	7.8	8.3	6.3	10.3
Israel	8.9	6.0	11.8	10.3	7.1	13.5	12.0	9.1	14.9	12.3	9.1	15.5	6.2	3.6	8.8	8.7	5.8	11.6
Italy	14.1	12.0	16.2	16.8	14.9	18.7	13.9	11.8	16.0	15.3	13.1	17.5	6.4	4.6	8.2	10.0	8.0	12.0
South Korea	21.9	20.1	23.7	22.8	21.1	24.5	28.0	26.3	29.7	27.9	26.2	29.6	12.0	10.5	13.5	13.8	12.3	15.3
Mexico	11.8	10.7	12.9	12.0	10.7	13.3	13.3	11.8	14.8	12.6	11.0	14.2	1.9	1.1	2.7	3.5	2.4	4.6
Netherlands	7.4	5.6	9.2	8.7	6.1	11.3	1.9	0.0	3.8	1.6	-1.0	4.2	-1.5	-3.0	0.0	-0.1	-2.5	2.3
Poland	8.6	5.0	12.2	9.7	6.2	13.2	10.7	7.1	14.3	10.5	7.0	14.0	3.2	0.0	6.4	5.7	2.2	9.2
Portugal	17.5	10.9	24.1	21.1	16.4	25.8	15.1	8.4	21.8	17.3	11.9	22.7	4.2	-1.8	10.2	7.4	2.5	12.3
Slovenia	5.5	0.4	10.6	7.2	4.2	10.2	11.5	6.3	16.7	12.9	9.8	16.0	4.1	-0.6	8.8	7.8	4.6	11.0
Spain	15.0	13.0	17.0	18.8	16.9	20.7	14.1	12.0	16.2	16.6	14.3	18.9	7.0	5.3	8.7	10.9	8.9	12.9
Sweden	10.9	8.8	13.0	11.7	9.6	13.8	5.9	3.7	8.1	5.4	3.2	7.6	-1.5	-3.6	0.6	0.3	-2.0	2.6
Switzerland	4.4	1.8	7.0	5.0	2.5	7.5	4.9	2.1	7.7	4.4	1.7	7.1	1.4	-0.8	3.6	3.1	0.9	5.3
United States	7.0	6.2	7.8	8.2	7.4	9.0	9.2	8.3	10.1	9.4	8.4	10.4	-0.3	-0.9	0.3	1.7	0.9	2.5

Notes: Predicted percent-point differences and 95% CIs based on models 2-4 (see Supplementary Tables 4-6).

Supplementary Table 14. Sociodemographic and economic characteristics at 2016 by country

Country	Population size (in millions)	GDP per capita (2015 USD)	Health expenditure (GDP %)	GINI index	Urban population (%)	Life expectancy, female	Life expectancy, male	Population ages 65+ (%)	Population 65+ growth (in percent points)	
									2006-2016	2000-2024
Austria	8.74	44,363	10.35	30.80	57.91	84.10	79.30	18.50	1.82	5.17
Belgium	11.33	41,173	10.79	27.60	97.92	84.00	79.00	18.36	1.20	3.75
China[1]	1,387.79	8,679	4.98	38.50	56.74	80.05	74.58	10.49	2.44	7.65
Czech Republic	10.57	18,359	7.39	25.40	73.57	82.10	76.10	18.60	4.34	7.08
Denmark	5.73	54,300	10.25	28.20	87.64	82.80	79.00	18.94	3.69	6.14
England[2]	65.61	45,486	9.77	33.10	82.89	83.00	79.40	17.92	1.99	6.05
Estonia	1.32	18,265	6.70	31.20	68.56	82.20	73.30	19.19	2.11	6.91
France	66.72	36,921	11.49	31.90	79.92	85.80	79.50	19.28	2.84	6.73
Germany	82.35	42,528	11.24	31.40	77.22	83.50	78.60	21.13	1.64	2.83
Greece	10.78	18,050	8.45	35.00	78.39	84.00	78.90	21.40	2.91	6.41
Israel	8.55	37,094	7.10	39.00	92.26	84.20	80.70	11.26	1.32	12.18
Italy	60.12	31,078	8.73	35.20	69.86	85.60	81.00	22.31	2.37	3.23
Korea	51.22	29,467	6.89	35.20	81.56	85.40	79.30	13.36	3.94	7.02
Mexico[1]	122.25	10,101	5.38	46.90	79.58	76.96	71.74	6.80	1.01	7.90
Netherlands	17.03	46,655	10.06	28.20	90.64	83.20	80.00	18.09	3.86	8.40
Poland	37.97	13,026	6.59	31.20	60.18	82.00	73.90	15.91	2.54	7.87
Portugal	10.33	19,663	9.39	35.20	64.09	84.30	78.10	21.03	3.56	3.48
Slovenia	2.07	21,310	8.48	24.80	54.02	84.30	78.20	18.51	2.91	4.69
Spain	46.46	26,719	8.95	35.80	79.84	86.30	80.50	18.77	2.23	5.74
Sweden	9.92	51,747	10.85	29.60	86.85	84.10	80.60	19.77	2.45	3.61
Switzerland	8.37	84,611	11.23	33.00	73.74	85.60	81.70	18.04	2.00	4.60
United States	324.35	57,431	16.79	41.30	81.86	81.10	76.10	14.61	2.43	6.30

[1]Country income classification according to World Bank Group: Upper-middle income (China, Mexico); High income (all others). [2]Great Britain. Source: World Development Indicators (Last accessed September 9, 2025).

Supplementary Figure 1. Overall pain social disparity index across continents, 2016 17

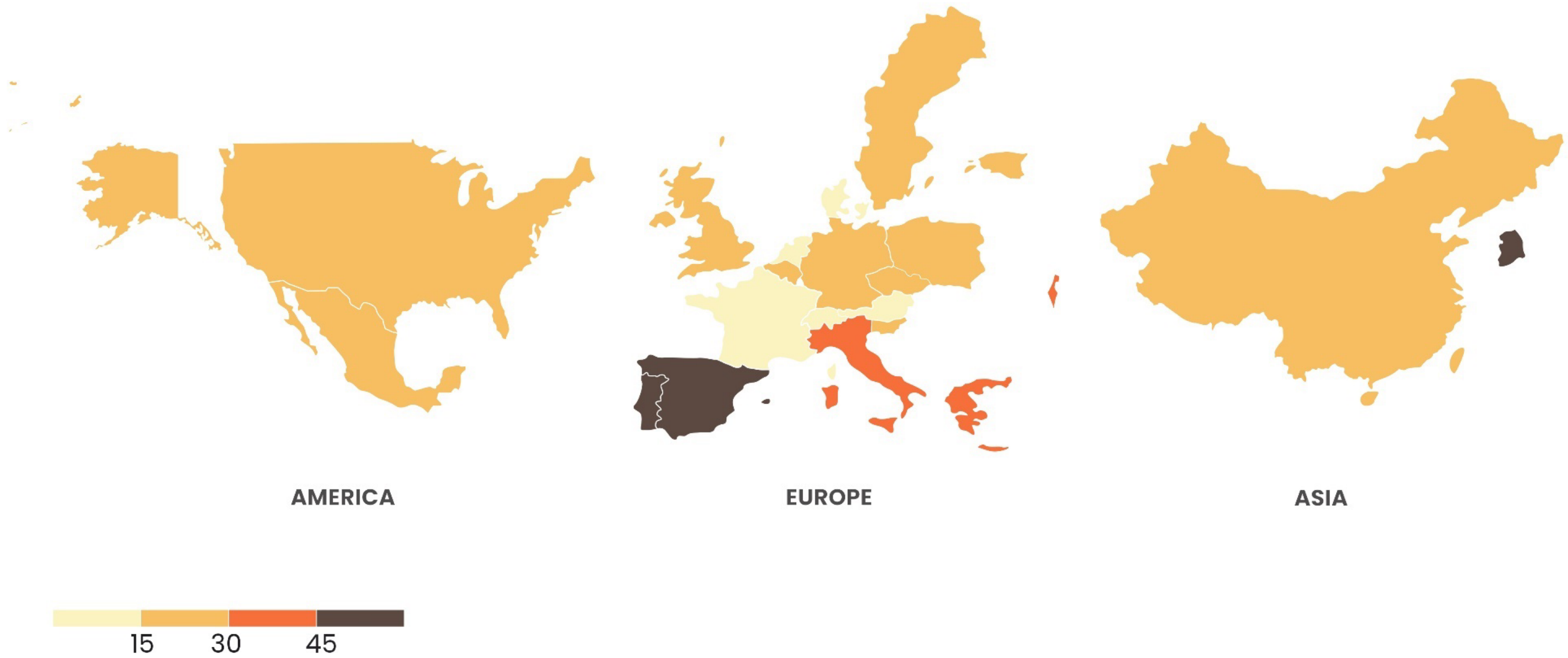

Note: The social disparity index is defined as the sum of three percentage point differences in pain prevalence in 2016: (1) female versus male, (2) less than high school completed versus high school completed or more, and (3) >60 years versus ages 50-60.

Supplementary Figure 2. Pain prevalence by risk group in 22 countries, 2006-2016

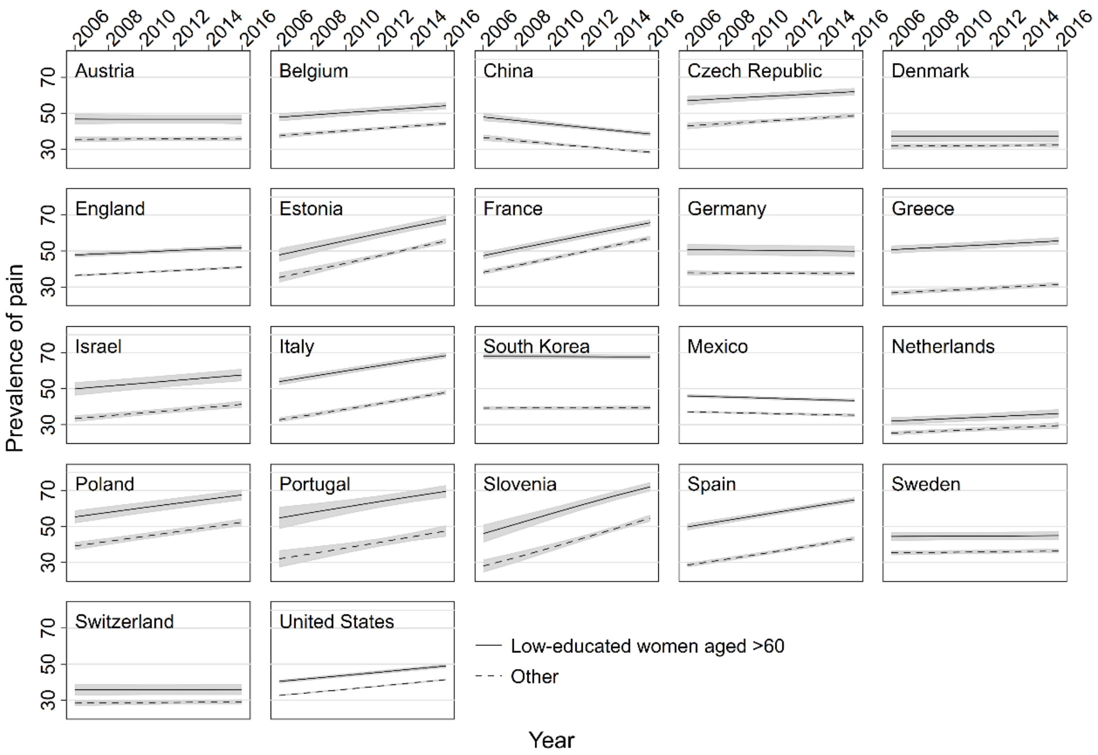

Notes: Predicted prevalences based on model 5, estimated for site-specific pain question and the high-risk pain group (female, less than high school completed, and over 60 years old; see Supplementary Table 7). The shaded grey area represents 95% confidence intervals.

Supplementary References

1. Blyth FM, Briggs AM, Schneider CH, Hoy DG, March LM. The Global Burden of Musculoskeletal Pain-Where to From Here? *Am J Public Health* 2019;**109**(1):35-40. (In eng). DOI: 10.2105/ajph.2018.304747.
2. Blyth FM, Huckel Schneider C. Global burden of pain and global pain policy-creating a purposeful body of evidence. *Pain* 2018;**159** Suppl 1:S43-S48. DOI: 10.1097/j.pain.0000000000001311
3. Briggs AM, Persaud JG, Deverell ML, et al. Integrated prevention and management of non-communicable diseases, including musculoskeletal health: a systematic policy analysis among OECD countries. *BMJ Global Health* 2019;**4**:e001806. DOI: 10.1136/bmjgh-2019-001806.
4. Miller GA, Chapman JP. Misunderstanding analysis of covariance. *J Abnorm Psychol* 2001;**110**:-8. doi: 10.1037/0021-843X.110.1.40. pmid: 11261398
5. Nicholas M, Vlaeyen JWS, Rief W, et al. The IASP classification of chronic pain for ICD-11: chronic primary pain. *Pain* 2019;**160**(1):28-37. DOI: 10.1097/j.pain.0000000000001390.
6. Routen A, Lekas HM, Harrison J, Khunti K. Intersectionality in health equity research. *BMJ*. 2023 Dec 29;**383**:2953. doi: 10.1136/bmj.p2953. PMID: 38158221. rt
7. Wang L, Ye H, Li Z, et al. Epidemiological trends of low back pain at the global, regional, and national levels. Eur Spine J 2022; **31**(4): 953-62.
8. Zenk SN, Simoni JM, Pérez-Stable EJ. Prioritizing Research on Social Determinants of Health-"Yes, and…". *JAMA Intern Med*. 2025 Mar 1;**185**(3):253-254. doi: 10.1001/jamainternmed.2024.6391. PMID: 39621326.
9. Zimmer Z, Zajacova A, Fraser K, Powers D, Grol-Prokopczyk H. A global comparative study of wealth-pain gradients: Investigating individual- and country-level associations. *Dialogues Health*. 2023 Dec;**2**:100122. doi: 10.1016/j.dialog.2023.100122.
